# Colon Cancer Stem Cells: Bench-to-Bedside—New Therapeutical Approaches in Clinical Oncology for Disease Breakdown

**DOI:** 10.3390/cancers3021957

**Published:** 2011-04-13

**Authors:** Simone Di Franco Simone, Pietro Mancuso, Antonina Benfante, Marisa Spina, Flora Iovino, Francesco Dieli, Giorgio Stassi, Matilde Todaro

**Affiliations:** 1 Department of Surgical and Oncological Sciences, Cellular and Molecular Pathophysiology Laboratory, Palermo, Italy; 2 Cellular and Molecular Oncology, IRCCS Fondazione Salvatore Maugeri, Pavia, Italy; 3 Division of Immunology and Immunogenetics, Department of Biotechnology and Medical and Forensic Biopathological (DIBIMEF), Palermo, Italy

**Keywords:** cancer stem cell, colorectal cancer (CRC), CD133, differentiation

## Abstract

It is widely accepted by the scientific community that cancer, including colon cancer, is a “stem cell disease”. Until a few years ago, common opinion was that all neoplastic cells within a tumor contained tumorigenic growth capacity, but recent evidences hint to the possibility that such a feature is confined to a small subset of cancer-initiating cells, also called cancer stem cells (CSCs). Thus, malignant tumors are organized in a hierarchical fashion in which CSCs give rise to more differentiated tumor cells. CSCs possess high levels of ATP-binding cassette (ABC) transporters and anti-apoptotic molecules, active DNA-repair, slow replication capacities and they produce growth factors that confer refractoriness to antineoplastic treatments. The inefficacy of conventional therapies towards the stem cell population might explain cancer chemoresistance and the high frequency of relapse shown by the majority of tumors. Nowadays, in fact all the therapies available are not sufficient to cure patients with advanced forms of colon cancer since they target differentiated cancer cells which constitute most of the tumor mass and spare CSCs. Since CSCs are the entities responsible for the development of the tumor and represent the only cell population able to sustain tumor growth and progression, these cells represent the elective target for innovative therapies.

## 1. Introduction

Colorectal cancer (CRC) is characterized by progressive mutations in oncogenes, or tumor suppressor genes: scientific evidences show that at least 4–5 mutations are necessary for a malignant tumor formation [1]. Some of these mutations seem to elapse often within the same sequences, and they are then shared by most individuals with this tumor, while others are different and therefore determine the final phenotype of cancer [2].

Most of the information on the mutations that cause CRC derived from studies on hereditary forms of cancer, making up 5% to 10% of all colon cancer cases. Familial adenomatous polyposis (FAP) is an autosomal dominant CRC syndrome caused by a mutation in the APC (adenomatous polyposis coli) gene which characterizes multiple CRC [3].

APC is at the base of the signaling pathway called wingless/wnt. The main function of APC is to modulate the cytoplasmic β-catenin levels, a protein that can migrate into the nucleus and activate transcription of protein complexes called TNA (transcription of cMyc and cyclin D1), responsible for the regulation of proliferation, differentiation, migration and apoptosis [4]. For subsequent progression, cancers need more mutations, including KRAS and TP53 and deletion on chromosome 18q [5].

According to the old paradigm of carcinogenesis, tumor cell population is heterogeneous and all neoplastic cells within a tumor have an equal capacity to proliferate and thereby to sustain tumor growth. Different from this notion, current evidences suggest that cancer growth is dictated by a small population called cancer stem cells. These cells have a self-renewal property and generate a progeny of non-tumorigenic cells. The latter gives rise to the non-tumorigenic differentiated population which represents the majority within tumor mass. Cancer stem cells can derive from either normal stem cells or progenitor cells as a consequence of genetic and/or epigenetic alterations [6]. Cancer stem cells commonly survive conventional treatment; even if therapy results in an apparent complete regression of primary tumor, remaining CSCs are able to induce the minimal residual disease (MRD). Therefore understanding the mechanism that maintains the immature state becomes crucial in order to develop new anti-tumor approaches.

## 2. Colonic Crypt Organization

The colon wall is composed of several layers: mucosa, submucosa, muscularis and serosa.

The mucosa consists mainly of two cell types: epithelial cells, with cylindrical shape, whose function is to reabsorb water and salts, and goblet mucipare cells, whose function is to secrete a slimy substance in the lumen, in order to lubricate the same and facilitate stool passage. The epithelial cells show on their outer surface, toward the lumen, a series of invaginations, called crypts of Lieberkuhn, which are designed to increase the absorbent surface. The submucosa lies immediately under the mucosa and is very rich in vascular structures, lymph and nerve fibers, that regulate peristalsis (intestinal propulsive movements that promote the progression of the stool toward the rectum). The muscolaris consists of two layers of muscle: an inner, cross-trending, and an outer longitudinal trending. They give the bowel a characteristic saccular appearance. The serosa, also called the peritoneum, constitutes an outer coating, covering the entire colon and also all other abdominal organs and viscera [6].

The epithelial layer presents about 14,000 crypts/square centimeter in the adult human colon, each of these crypts contains 2,000 to 3,000 cells, and the colonic stem cells are located at the base, surrounded by mesenchymal cells to form the stem niche [5-11]. Each crypt in the intestine is mainly composed of three different cell types: the colonocytes or columnar cells, the mucin-secreting goblet cells and the endocrine cells. All these cells are generated starting from a colonic stem cell that, via asymmetric division, can generate a cell identical to itself (self-renewal capacity), and a transit cell that can proliferate and differentiate by migrating up to the top of the crypt. This “unitarian theory” (one single cell can generate all the cell types) was first formulated in 1974 [12] and later experimentally demonstrated [13,14]. These stem cells are responsible for the perpetual turn-over of the colonic epithelial cells during the whole lifetime of an individual. There is a continuous supply of these cells, every 2–7 days under normal conditions, and an increased turnover of them in tissue damage conditions.

The complexity of the crypt structure was an obstacle in understanding the key mechanisms that lead to the formation of the crypt from a single stem cell. The first studies to identify the colonic stem cell population were based on Chang *et al.* studies by using ^3^H-thymidine injection [15], and recently confirmed by bromodeoxyuridine DNA-labeling dye [16] for slow-cycling stem cells localization.

There are two models regarding the positioning of the stem cells: the “stem cell zone” model, and the “+4 position” model. The “stem cell zone” model describes the colon stem cells, the crypt base columnar cells (CBC), at the very bottom of the crypts. On the contrary, the “+4 position” model, related to the intestinal crypt, claims that the intestinal stem cells are located at the +4 position above the Paneth cells at the base of the crypt [17]. Actually the absence of specific colonic stem cell markers makes their identification and positioning rather difficult.

Adult stem cells are defined by two fundamental properties: self-renewal and differentiation capacity to generate all the cyto-types of that tissue. An important aspect in studying stem cells is the mechanism of cell division: stem cells seem to divide more slowly than the progenitor cells and differentiated cells [18]. Stem cells may undergo asymmetric division, thus generating two different cells, one stem cell identical to the mother cell, and a specialized one; but they can also make symmetrical division, generating two identical stem cells. The asymmetric division is slower and ensures the persistence of a pool of adult stem cells, and through cell differentiation, the continuous regeneration of organs and tissues [19]. According to the cell type division, it is possible to obtain a “lineage expansion” if stem cells are generated, or “lineage extinction” if differentiated cells are propagated [20]. The idea is widely accepted that the stem cells are responsible for giving rise to cancer, just because their slow cycles of division and longevity of life allow them to accumulate different mutations over time that could lead to so-called cancer stem cells [18].

## 3. Intestinal Niche

**Figure 1.**
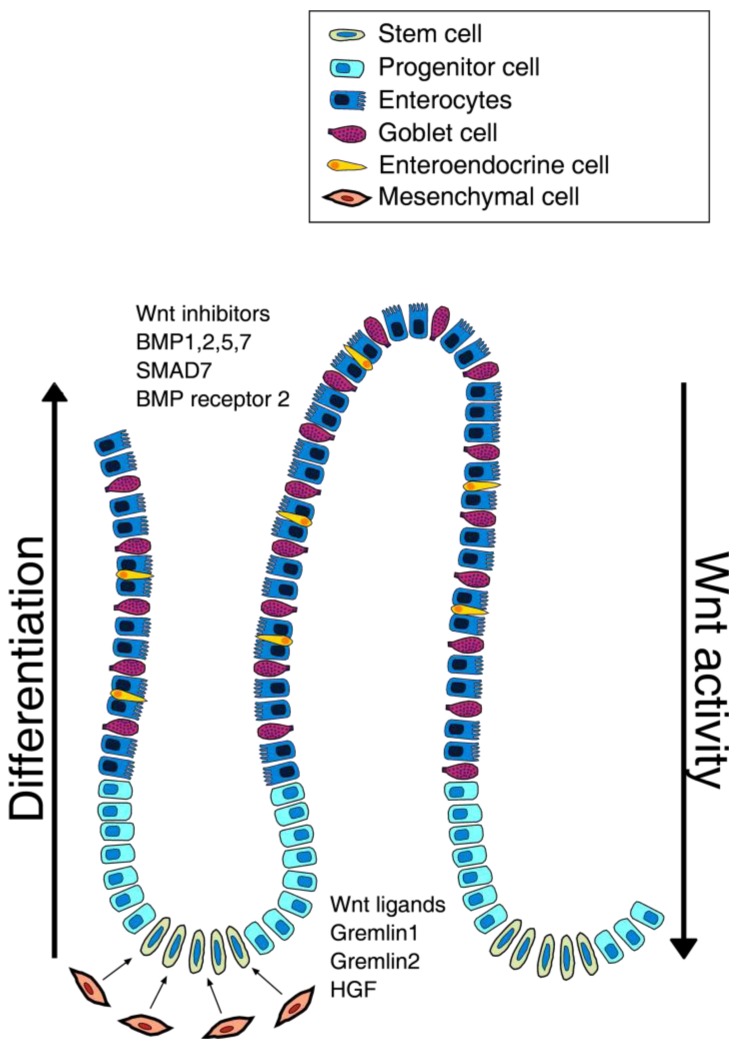
Graphic representation of a colon crypt. This image shows the distribution of different cell types along the colon crypt unit. At the base of the crypt the mesenchymal cells (ISEMFs) are represented and the factors responsible for the stem cell niche maintenance. The progressive cellular differentiation toward the villus apex is also shown, where many factors that inhibit Wnt activity are over-expressed.

The intestinal niche is defined as the environment responsible for stem cells maintenance that is controlled by fine signals that ensure stem cells proliferation. The most determining effect seems to be due to the population of intestinal sub-epithelial myofibroblasts (ISEMFs), whose role is to regulate the organogenesis and tissue repair, and whose growth appears to be regulated by several growth factors [21,22]. Recent findings show that maintenance of stem niche is controlled by Wnt, Bone Morphogenetic Protein (BMP), Notch and Sonic hedgehog (Shh) pathways (Figure 1).

In this signal network, the Wnt pathway definitely has a key role: the central role is played by β-catenin, that, in the absence of Wnt ligands, binds the APC protein, the glycogen synthase kinase 3β (GSK3β) and axin, to be then phosphorylated, ubiquitinated and finally degraded by the proteasome machinery [23]. Instead, Wnt activation requires the binding of Wnt family proteins to their receptors of the Frizzled family (Fz) that subsequently promotes β-catenin accumulation into the nucleus, which binds TCF4, activating the transcription of several genes involved in cell cycle regulation and proliferation [24]. β-catenin also induces the expression of Ephrin receptors EphB1 and EphB2, which regulate stemness maintenance, cell migration and differentiation [25]: these receptors, following interaction with ephrin ligands, extend the cell proliferation domain in areas higher up the crypts [26].

Interestingly, Wnt pathway members are differently distributed along the axis of the crypt *i.e.* the mRNA for secreted Fz-related protein (sFRP)-5, Wnt-3, Wnt-6, Wnt-9b and Fz-5 were found at the base of the crypts, with decreasing concentration towards the apex of the crypts where more differentiated cells reside. Moreover the cells at the top of the crypt seem to express Wnt inhibitor factors [27].

Recently, Vermeulen *et al.* [29] have demonstrated the important effect of myofibroblasts and the factors secreted by them, such as the hepatocyte growth factor (HGF) in maintaining the stem cell niche of the intestinal crypts. Differentiated cells are able to revert to their phenotype, reverting to stem cells (tumorigenic) in response to the addition of myofibroblasts or HGF.

In addition to Wnt, BMP, Notch and Shh pathways play crucial roles in niche homeostasis. BMP proteins are a subset of the TGF-β super-family members that, after linking their receptors, trigger different biological processes [28]. This pathway leads to the phosphorilation of Smad1, Smad5, Smad8/R-Smad [30], that together with Smad4 (co-Smad), move to the nucleus, and in cooperation with other transcription factors, can regulate the target genes expression [31]. It was recently demonstrated that BMP promotes terminal differentiation and apoptosis, increasing the conventional therapeutic activity in tumors that do not show concomitant mutation of SMAD4 and constitutive activation of PI3K [32].

Moreover, Kosinski *et al.* [33] demonstrated that there is a precise distribution of the different factors along the crypt: at the apex of the crypt the cells express high levels of BMP1, BMP2, BMP5, SMAD7, BMP7, and BMP receptor 2, while cells at the base of the crypt, probably due to the presence of myofibroblasts, produce high levels of BMP antagonists as GREM1, GREM2 and chordin-like-1, which contribute to the maintenance of stemness.

Notch pathway is one of the most studied cell signaling systems that includes four different type I trans-membrane receptor: Notch1, Notch2, Notch3 and Notch4. Its activation involves the binding of five different ligands including Jagged-1 (JAG1), -2 (JAG2), Delta-like (DLL) 1, 2 (DLL2) and 4 (DLL4): the extracellular binding of these ligands triggers the release of the intracellular domain (NICD) through proteolytic cleavage mediated by some metallo-proteases, such as ADAM10 or ADAM17. The NICD moves to the nucleus where it forms a complex with some DNA-binding proteins, converting them from inhibitors to activators of all the target genes transcription [34].

Finally, Sonic hedgehog (Shh) plays an important role during gut organogenesis. The activation requires Shh binding to its receptor, Patched (PTCH), which allows the release of the G-coupled protein Smoothened (SMO) that, together with the GLI transcription factors, migrates into the nucleus inducing target genes activation [35].

## 4. Cancer Stem Cell Theory

The idea that cancer is composed of a morphologically heterogeneous population of cells, differing in markers expression, proliferation capacity and tumorigenicity, has been described more than a century ago [2,36-38]. It is widely recognized that this heterogeneity is caused by genetic/epigenetic hits and micro-environmental differences that determine several degrees of cell differentiation [39]. In recent years, novel insights in cancer research have suggested that the capacity to initiate and sustain tumor growing is a unique characteristic of a small subset of cancer cells with stemness properties within the tumor mass, called “cancer stem cells” (CSCs) or “tumor-initiating cells” [40].

This discovery has profoundly changed the way to look at cancer, which has previously only been seen as a genetic disease [41]. There are two different models of cancer that could explain the development of tumor: the first one, the “classic model” of tumorigenesis, postulated by Vogelstain and Nowell [2,36], describes the tumor development through sequential mutations in oncogenes and tumor suppressor genes. According to this theory, tumors consist of a heterogeneous cell population that, acquiring new mutations, undergoes uncontrolled proliferation and invasivity. This stochastic model considers all cancer cells able to reform a tumor, after implantation in immuno-compromised mice [42]. Contrarily the second theory, the “cancer stem cells” model, is based on evidence that only a small subset of cells, the CSCs, within the tumor population, can initiate and sustain tumor growth [43].

Emerging evidences suggest that CSCs, isolated from a variety of tumor types, retain tumorigenic capacity and are responsible for the propagation, relapse and metastatic dissemination. CSCs are defined by sharing stem cell-like features with the normal stem cells, such as self-renewal and pluripotent differentiation capacity. CSCs could derive from self-renewing of normal cells after genetic/epigenetic changes, or from progenitor cells that acquire self-renewal capacity. The link between cancer and normal stem cells has also been demonstrated on the basis of common signaling pathways that regulate self-renewal, including Wnt, Notch and Sonic Hedgehog (Shh): the deregulation of these pathways plays a key role in the tumorigenesis process [44]. Many studies have shown the importance of self renewal pathway activation for CSCs maintenance [45]. Jamieson and colleagues [46] first identified the aberrant Wnt/βcatenin self renewal pathway activation in leukemic stem cell propagation; Wnt pathway has been later considered important also in breast cancer stem cells (BCSCs). Korkaya *et al.* [47] showed that the increased activity of Wnt/β-catenin was mediated by activation of Akt signaling activation. Defects in Notch pathway, normally implicated in stem cell growth and differentiation, have been seen in the colon CSC (CCSC) subset. It was observed that using antibody anti DLL4, an important component of Notch pathway, the growth of human colon cancer xenograft was inhibited, directly inhibiting Notch signaling. Notch pathway is also activated in breast [48] and glioblastoma CSCs model. Finally, alterations in Hedgehog signaling pathway, have been reported in many tumors: leukemia [49,50], pancreatic, gastric, prostate, breast [51,52], glioblastoma [53] and colon cancer [54].

The discovery of CSCs has changed the view of carcinogenesis and therapeutic approaches over recent years. Tumors are considered to be able to evade death signals induced by therapeutic drugs through multiple mechanisms, even if the molecular bases concerning the failure of chemotherapy have not yet been defined. The CSCs are characterized by high resistance to drugs and general toxins, which target rapidly proliferating cells and spare the slow dividing cells, due to an up-regulation of several ATP-binding cassette transporters, active DNA-repair capacity, over-expression of anti-apoptotic molecules that cause changes in the signaling pathways controlling proliferation, differentiation and apoptosis [55].

The first CSCs were isolated from acute myeloid leukemia (AML) and then characterized by the presence of immature cells, the blasts, detected in blood and bone marrow by John Dick and colleagues [56,57]. They have indeed isolated a sub-population of CD34^+^ CD38^-^ leukemic stem cell from patients with AML and they observed that just a small number of leukemic cells were able to form colonies growing *in vitro*. They have also found that there was a sort of hierarchy in leukemic cells and that only CD34^+^ CD38^-^ cells, if transplanted into immunodeficient mice, were able to reproduce the parental tumor phenotype [55]. Using similar approaches, many types of tumor stem cells have been identified from a variety of solid tumors. In particular Al Hajj *et al.* [58] showed that CD44^+^/CD24^-^ cell population was enriched in breast cancer stem cells (BCSCs). After the publications about leukemia and breast cancer, many reports showed how to isolate the CSCs in several malignancies including: brain [59], colon [60-62], head and neck [63], pancreas [64,65], melanoma [66], mesenchymal [67], hepatic [68], lung [69], prostate [70], and ovarian [71] tumors.

Despite several scientific evidences about CSCs existence, there is still an alternative theory sustaining that this cell population would not behave as an entity, but as a phenotypic state, which was observed in stem cells of melanoma [72] as well as during epithelial-mesenchymal transition [73], where stem cells acquire stemness properties.

## 5. Colon Stem Cell Markers

Stem cells characterization is yet unclear even if several molecules have been identified as putative stemness markers because none are considered exclusive. There are indeed important debates about the value of each marker: scientific evidences have shown that it is possible to obtain a cell population enriched in colon stem cells through cell sorting, using different combinations of markers (Table 1) [62].

Msi-1 is an RNA-binding protein, it was one of the first molecules studied as a colon stem cell marker and its role was mostly studied in *Drosophila Melanogaster*, where it seems to be essential in the mechanisms of asymmetric cell division that regulate neural development [74]. It is also considered fundamental in the development of the nervous system of mammals [75]. Its location in murine and human small intestine, at the base of the crypts, makes it very important in the characterization of colon stem cells.

Among all cell surface putative stemness markers, β_1_ integrin (CD29), reported as a marker of high-proliferation, was found at high expression levels at the base of the crypts, detecting both stem cells and progenitor cells [76]. According to these data, the EphB receptors expression consists in a gradient with the highest levels at the base of the crypts, and lower ones at the crypt-villus junction [77,78].

Bmi-1, a repressor of the Polycomb group, was found essential for self-renewal of hematopoietic stem cells and adult neural stem cells, through repression of genes involved in senescence, suggesting that stem cells developed specific mechanisms to extend their proliferative capacity. It is indeed expressed in the small intestine near to the crypt's bottom, in line with the idea that this zone is the residence of colon stem cells [79]. Bmi-1 is over-expressed in patients and results in very poor survival [80].

DCAMKL-1 is proposed as a putative colon stem marker: it is a microtubule-associated kinase that can undergo auto-phosphorylation. DCAMKL-1^+^ cells are resistant to apoptosis after ionizing radiation injury.

More recently Lgr5 protein (Gpr49), a G protein-coupled receptor, whose gene is a Wnt regulation target, has been recently studied as an elective colorectal stem marker, even if its function remains unclear. It was demonstrated, in agreement with the multi-lineage capacity, that a single Lgr5^+^ cell is able to generate a whole crypt-like structure *in vitro*, generating any cell type present in the colonic epithelium [81]. Recently some reports showed Lgr5 over-expression in advanced CRCs and its correlation with cancer progression [82].

The first direct evidence supporting the CSC hypothesis came from the recent finding of self-renewal and tumor-initiating cells with a common and distinct surface-expressed polypeptide, the CD133 pentaspan trans-membrane glycoprotein, also known as Prominin-1. This protein was first released as a marker for hematopoietic stem cells and progenitor cells and it was subsequently used to identify many tumors [83]: brain [59], prostate, hepatocellular and colon tumors [60,61,84,85].

The stemness value of CD133 has been much debated, in particular the tumorigenic potential of colon CD133^+^ cells and the ability of these cells to give rise to a tumor in NOD-SCID mice. Many research groups showed that only the CD133^+^ cells within a colon carcinoma are able to initiate and sustain tumor growth [77,78,89,90].

CD133^+^ cells are maintained in culture for a long time without losing their ability to reproduce the parental human phenotype: CCSCs, after enzymatic digestion, can be expanded as tumor spheroids *in vitro* with a serum-free medium complemented with epidermal growth factor (EGF) and basic fibroblast growth factor (bFGF), using low adhesion conditions (to induce differentiated cells death due to the *anoikis*) [86]. In differentiation conditions CD133^+^ cells are able to generate particular structures similar to crypt; moreover, during *in vitro* or *in vivo* differentiation these cells gradually acquire typical colon epithelial markers, such as CK20, and at the same time decrease CD133 expression. According to these findings much clinical data identify CD133 as an independent negative prognostic marker [87]; its expression in combination with nuclear β-catenin is very important to determine poor patient survival [88,89].

Since the use of one single marker it is considered insufficient for the identification and isolation of CCSCs, many researchers usually perform a sorting using several different putative markers. Dalerba *et al.* [62] showed that CD133^+^ cells express the stem-like epithelial specific antigen (EpCAM), CD44 and CD166. In their study the injection of CD44^+^ and EpCAM^hi^ cells, into NOD-SCID mice, reproduced a tumor xenograft phenotypically similar to parental one.

Supporting this thesis, Du's group [90] has shown that CD44 could be considered as an important marker in CCSCs that give rise to spheres *in vitro* and to a xenograft similar to the original tumor *in vivo*. More recently, aldehyde dehydrogenase-1 (ALDH1), a detoxifying enzyme, has been proposed as a marker to identify, isolate and track, human CSCs during CRC development [91].

The possibility to isolate and to study CSCs represents a revolutionary approach in cancer research to better understand the pathogenesis of cancer, so these cells are an elective target for new therapies.

## 6. Alternative and Synergistic Therapies

Today most of the existing conventional therapies are insufficient to permanently eradicate the tumor or to treat patients with advanced forms of CRC. Almost all colon cancers begin as benign polyps that can slowly develop into malignant tumors.

To timely remove precancerous polyps, before malignant transformation and subsequent metastasization (the liver is the most common site), would be appropriate especially for patients with familiarity. Preventive colonoscopy could lead to the the surgical removal of the cancer as soon as possible.

Nowadays, for patients with metastatic CRC to the liver, there are two useful treatments available, FOLFOX (Folinic acid/Fluorouracil and Oxaliplatin) and FOLFIRI (FOLFOX plus vitamin B and irinotecan). Sometimes Cetuximab, a monoclonal antibody, is added to FOLFIRI [92]. FOLFOX and FOLFIRI have demonstrated good efficacy in Phase III trials and are actually employed more frequently in younger than in older patients with metastatic CRC [93]. Neoadjuvant chemotherapy has been combined with anti-angiogenic drugs in metastatic colon cancer patients, treated with Bevacizumab, a humanized monoclonal antibody that targets the vascular endothelial growth factor (VEGF) [94], which is an important angiogenic factor in primary and metastatic human CRC [95]. VEGF expression is observed early in the progression from premalignant adenoma to invasive and metastatic disease. Additionally, VEGF expression has been correlated with increased micro-vessel count in colon tumors, and both VEGF and micro-vessels count have been associated with poor outcomes, as measured by tumor size, metastasis and patient survival. Another neo-adjuvant drug for colorectal cancer is Cetuximab, also known as Erbitux, a monoclonal antibody that inhibits the epidermal growth factor receptor (EGFR), involved in cell differentiation and proliferation [92]. Cetuximab is indicated for the treatment of EGFR expressing patients, KRAS wild-type metastatic colorectal cancer, alone or in combination with FOLFIRI. Two large clinical studies of cetuximab, OPUS and CRYSTAL, have recently been published, and have provided further evidence that cetuximab significantly improves response rates and disease-free survival in metastatic CRC patients with KRAS wild-type tumors [92]. New targeted therapies under investigation are directed not only against downstream factors of the EGFR pathway, but also toward correlated pathways, to overcome growth factor–mediated resistance.

An alternative therapy could selectively target CSCs pathways such as IL-4, that is a cytokine produced in an autocrine way by CCSCs; it is known for its involvement in activated B-cell stimulation, T-cell proliferation and the differentiation of CD4^+^ T-cells into Th2 cells [96].

In CCSCs, the inhibition of IL-4 signaling transduction pathway with anti–IL-4 neutralizing antibody or IL-4 receptor α antagonist, leads to the sensibilization of these cells to chemotherapeutic agents through down-regulation of anti-apoptotic proteins, such as cFLIP, Bcl-xL, and PED. IL-4 antibodies treatment, in combination with standard chemotherapeutic agents (5-fluorouracil or oxaliplatin) reduces tumor growth: this phenomenon is confirmed also *in vivo* where this treatment significantly reduces xenograft tumors growing [85]. Recent studies have demonstrated that the up-regulation of IL-4 cytokine in CD133^+^ CCSCs stem cells is an important mechanism that protects these tumorigenic cells from apoptosis [97].

BMP4 is another important molecule because of its ability to activate a differentiation program and stimulate apoptosis in CCSCs, reducing β-catenin activation through inhibition of PI3K/AKT pathway and up-modulation of Wnt-negative regulators. Also in this case chemotherapeutic agents, such as oxaliplatin and 5-flourouracil, increase the anti-tumor activity of BMP4 since their concomitant administration induces complete long-term regression of colon CSC-derived xenograft tumors [32].

Cancer immunotherapy could be considered an important approach taking advantage of the forcefulness and specificity of the immune system. Although cancer cells are less immunogenic than their normal counterpart, the immune system is clearly able to recognize and eliminate them. Thus, the challenge for immunotherapy is to use advances in cellular and molecular immunology to develop strategies that effectively and safely increase antitumor responses [98].

Most cancers are resistant to current therapies due to the slow-cycling CSCs and because of the location of these cells within hypoxic niches [99,100]. Clinical studies have demonstrated that, in terms of survival, the synergic use of chemotherapy and immunotherapy greatly benefited the health of the patient compared to chemotherapy alone [101]. Chemotherapeutic agents can also stimulate tumors to immune cell-mediated killing, increasing sensitivity of tumor cells to cytotoxicity through T cells across the up-regulation of death receptors Fas and TRAIL-R2 (DR5) ligands to FasL (CD95L) and TRAIL, respectively [102].

Most current immunotherapeutic approaches aim at inducing antitumor response sensitizing the adaptive immune system, depending on MHC-restricted αβ T cells. Anyway, in cancer cells, loss of MHC molecules is recurrently observed, making tumor cells resistant to αβ T cell-mediated cytotoxicity. γδ T cells show potent MHC-unrestricted lytic activity *versus* different tumor cells *in vitro*, suggesting their potential employment in anticancer therapy. Moreover, γδ T cells have been isolated and identified from tumor infiltrating lymphocytes in different cancer types, including prostate carcinoma [103]. Antigen recognition of γδ T-cell receptors is strictly selective and the responses frequently exhibit native characteristics. Furthermore peripheral γδ T cells exert several regulatory functions, rapidly producing cytokines, such as interferon (IFN)-γ and IL-17, and they also promote inflammation. Nevertheless, γδ T cells improve tumor clearance, directly through target cell lysis. The fruitful interaction of γδ T-cell and other immune cells may be critical for immune regulation and host defense [104]. Moreover, the incubation of the CCSCs with bisphosphonate zoledronate leads to a relevant γδ T-cell response against different tumor cells *in vitro*, even if this experiment represented the first report in employing γδ T cell to target CSCs [103].

All the therapies mentioned above should be validated in order to avoid survival of CSCs responsible for tumor recurrences.

## 7. Concluding Remarks

CSCs might derive from normal stem cells or SC-like progenitor cells that acquire genetic/epigenetic hits necessary for tumorigenesis; they also retain important biological features in common with normal stem cells, such as self-renewal and pluripotent capacities. Many self-renewal pathways undergo deregulation during neoplastic development. Moreover, CSCs' plurypotency properties support the idea that a tumor is an aberrantly developed organ, constituted by a heterogeneous cell population.

The role of CSCs in CRC is gaining interest, since this hypothesis could explain carcinogenesis, helping to define innovative therapeutic strategies focused on the tumorigenic sub-population. The highly negative prognosis of CRC is due to the inefficacy of current treatments in definitively eradicating the tumor. Accordingly, tumor growth/progression arrest requires the targetted elimination of CSCs considered responsible for minimal residual disease in order to prevent recurrences and metastasization.

## Figures and Tables

**Table 1.** Panel representing putative stemness markers.

**Putative marker**	**Alternative name**	**Roles**	

CD34		Stemness maintenance	Normal colon
DCAMKL1		Kinase, resistance to apoptosis	
EphB receptors		Stemness maintenance, cell migration	
Msi-1		RNA-binding protein, asymmetric division	

Bmi-1		Polycomb group repressor, self-renewal, senescence inhibitor	Colorectal cancer
CD24	HSA	Cell adhesion molecule	
CD29	β1 Integrin	Proliferation, matrix-cell interaction	
CD44		Cell-cell interaction, hyaluronic acid receptor, cell migration	
CD133	Prominin1	Self-renewal, tumorigenesis	
CD166	ALCAM	Cell adhesion molecule	
ESA	EpCAM	Cell adhesion molecule	
Lgr-5	Gpr49	G protein-coupled receptor, unclear function	
ALDH1		Detoxifying enzyme	
nuclear β-catenin		Cell cycle regulation, proliferation	

List of putative stem cell markers and their specific roles.

## References

[1] Vogelstein B., Fearon E.R., Hamilton S.R., Kern S.E., Preisinger A.C., Leppert M., Nakamura Y., White R., Smits A.M., Bos J.L. (1988). Genetic alterations during colorectal-tumor development. N. Engl. J. Med..

[2] Fearon E.R., Vogelstein B. (1990). A genetic model for colorectal tumorigenesis. Cell.

[3] Galiatsatos P., Foulkes W.D. (2006). Familial adenomatous polyposis. Am. J. Gastroenterol..

[4] Fearnhead N.S., Britton M.P., Bodmer W.F. (2001). The ABC of APC. Hum. Mol. Genet..

[5] Kinzler K.W., Vogelstein B. (1996). Lessons from hereditary colorectal cancer. Cell.

[6] Barker N., Ridgway R.A., van Es J.H., van de Wetering M., Begthel H., van den Born M., Danenberg E., Clarke A.R., Sansom O.J., Clevers H. (2009). Crypt stem cells as the cells-of-origin of intestinal cancer. Nature.

[7] Booth C., Potten C.S. (2000). Gut instincts: Thoughts on intestinal epithelial stem cells. J. Clin. Invest..

[8] Potten C.S., Loeffler M. (1990). Stem cells: Attributes, cycles, spirals, pitfalls and uncertainties. Lessons for and from the crypt. Development.

[9] Brittan M., Wright N.A. (2002). Gastrointestinal stem cells. J. Pathol..

[10] Potten C.S., Kellett M., Roberts S.A., Rew D.A., Wilson G.D. (1992). Measurement of *in vivo* proliferation in human colorectal mucosa using bromodeoxyuridine. Gut.

[11] Boman B.M., Huang E. (2008). Human colon cancer stem cells: A new paradigm in gastrointestinal oncology. J. Clin. Oncol..

[12] Cheng H., Leblond C.P. (1974). Origin, differentiation and renewal of the four main epithelial cell types in the mouse small intestine. V. Unitarian Theory of the origin of the four epithelial cell types. Am. J. Anat..

[13] Paulus U., Potten C.S., Loeffler M. (1992). A model of the control of cellular regeneration in the intestinal crypt after perturbation based solely on local stem cell regulation. Cell Prolif..

[14] Kirkland S.C. (1988). Clonal origin of columnar, mucous, and endocrine cell lineages in human colorectal epithelium. Cancer.

[15] Chang W.W., Leblond C.P. (1971). Renewal of the epithelium in the descending colon of the mouse. I. Presence of three cell populations: Vacuolated-columnar, mucous and argentaffin. Am. J. Anat..

[16] Kim S.J., Cheung S., Hellerstein M.K. (2004). Isolation of nuclei from label-retaining cells and measurement of their turnover rates in rat colon. Am. J. Physiol. Cell Physiol..

[17] Ricci-Vitiani L., Fabrizi E., Palio E., De Maria R. (2009). Colon cancer stem cells. J. Mol. Med..

[18] McDonald S.A., Preston S.L., Lovell M.J., Wright N.A., Jankowski J.A. (2006). Mechanisms of disease: From stem cells to colorectal cancer. Nat. Clin. Pract. Gastroenterol. Hepatol..

[19] Potten C.S., Booth C., Pritchard D.M. (1997). The intestinal epithelial stem cell: The mucosal governor. Int. J. Exp. Pathol..

[20] Kim K.M., Calabrese P., Tavare S., Shibata D. (2004). Enhanced stem cell survival in familial adenomatous polyposis. Am. J. Pathol..

[21] Adegboyega P.A., Mifflin R.C., DiMari J.F., Saada J.I., Powell D.W. (2002). Immunohistochemical study of myofibroblasts in normal colonic mucosa, hyperplastic polyps, and adenomatous colorectal polyps. Arch. Pathol. Lab. Med..

[22] Powell D.W., Mifflin R.C., Valentich J.D., Crowe S.E., Saada J.I., West A.B. (1999). Myofibroblasts. II. Intestinal subepithelial myofibroblasts. Am. J. Physiol..

[23] Reya T., Clevers H. (2005). Wnt signalling in stem cells and cancer. Nature.

[24] He T.C., Sparks A.B., Rago C., Hermeking H., Zawel L., da Costa L.T., Morin P.J., Vogelstein B., Kinzler K.W. (1998). Identification of c-MYC as a target of the APC pathway. Science.

[25] Crosnier C., Stamataki D., Lewis J. (2006). Organizing cell renewal in the intestine: Stem cells, signals and combinatorial control. Nat. Rev. Genet..

[26] Holmberg J., Genander M., Halford M.M., Anneren C., Sondell M., Chumley M.J., Silvany R.E., Henkemeyer M., Frisen J. (2006). EphB receptors coordinate migration and proliferation in the intestinal stem cell niche. Cell.

[27] Gregorieff A., Pinto D., Begthel H., Destree O., Kielman M., Clevers H. (2005). Expression pattern of Wnt signaling components in the adult intestine. Gastroenterology.

[28] Chen D., Ji X., Harris M.A., Feng J.Q., Karsenty G., Celeste A.J., Rosen V., Mundy G.R., Harris S.E. (1998). Differential roles for bone morphogenetic protein (BMP) receptor type IB and IA in differentiation and specification of mesenchymal precursor cells to osteoblast and adipocyte lineages. J. Cell Biol..

[29] Vermeulen L., De Sousa E Melo F., van der Heijden M., Cameron K., de Jong J.H., Borovski T., Tuynman J.B., Todaro M., Merz C., Rodermond H., Sprick M.R., Kemper K., Richel D.J., Stassi G., Medema J.P. (2010). Wnt activity defines colon cancer stem cells and is regulated by the microenvironment. Nat. Cell Biol..

[30] Miyazono K. (2000). TGF-beta signaling by Smad proteins. Cytokine Growth Factor Rev..

[31] Derynck R., Zhang Y.E. (2003). Smad-dependent and Smad-independent pathways in TGF-beta family signalling. Nature.

[32] Lombardo Y., Scopelliti A., Cammareri P., Todaro M., Iovino F., Ricci-Vitiani L., Gulotta G., Dieli F., De Maria R., Stassi G. (2011). Bone Morphogenetic Protein 4 Induces Differentiation of Colorectal Cancer Stem Cells and Increases Their Response to Chemotherapy in Mice. Gastroenterology.

[33] Kosinski C., Li V.S., Chan A.S., Zhang J., Ho C., Tsui W.Y., Chan T.L., Mifflin R.C., Powell D.W., Yuen S.T., Leung S.Y., Chen X. (2007). Gene expression patterns of human colon tops and basal crypts and BMP antagonists as intestinal stem cell niche factors. Proc. Natl. Acad. Sci. USA.

[34] Dikic I., Schmidt M.H. (2010). Notch: Implications of endogenous inhibitors for therapy. Bioessays.

[35] Hegde G.V., Munger C.M., Emanuel K., Joshi A.D., Greiner T.C., Weisenburger D.D., Vose J.M., Joshi S.S. (2008). Targeting of sonic hedgehog-GLI signaling: A potential strategy to improve therapy for mantle cell lymphoma. Mol. Cancer Ther..

[36] Nowell P.C. (1976). The clonal evolution of tumor cell populations. Science.

[37] Weiss L. (2000). Metastasis of cancer: A conceptual history from antiquity to the 1990s. Cancer Metastasis Rev..

[38] Hope K.J., Jin L., Dick J.E. (2004). Acute myeloid leukemia originates from a hierarchy of leukemic stem cell classes that differ in self-renewal capacity. Nat. Immunol..

[39] Alison M.R., Lim S.M., Nicholson L.J. (2011). Cancer stem cells: Problems for therapy?. J. Pathol..

[40] Clarke M.F., Dick J.E., Dirks P.B., Eaves C.J., Jamieson C.H., Jones D.L., Visvader J., Weissman I.L., Wahl G.M. (2006). Cancer stem cells-perspectives on current status and future directions: AACR Workshop on cancer stem cells. Cancer Res..

[41] Vermeulen L., Sprick M.R., Kemper K., Stassi G., Medema J.P. (2008). Cancer stem cells old concepts, new insights. Cell Death Differ..

[42] Al-Hajj M., Clarke M.F. (2004). Self-renewal and solid tumor stem cells. Oncogene.

[43] Dick J.E. (2003). Stem cells: Self-renewal writ in blood. Nature.

[44] Dontu G., Jackson K.W., McNicholas E., Kawamura M.J., Abdallah W.M., Wicha M.S. (2004). Role of Notch signaling in cell-fate determination of human mammary stem/progenitor cells. Breast Cancer Res..

[45] Yilmaz O.H., Valdez R., Theisen B.K., Guo W., Ferguson D.O., Wu H., Morrison S.J. (2006). Pten dependence distinguishes haematopoietic stem cells from leukaemia-initiating cells. Nature.

[46] Jamieson C.H., Ailles L.E., Dylla S.J., Muijtjens M., Jones C., Zehnder J.L., Gotlib J., Li K., Manz M.G., Keating A., Sawyers C.L., Weissman I.L. (2004). Granulocyte-macrophage progenitors as candidate leukemic stem cells in blast-crisis CML. N. Engl. J. Med..

[47] Korkaya H., Paulson A., Charafe-Jauffret E., Ginestier C., Brown M., Dutcher J., Clouthier S.G., Wicha M.S. (2009). Regulation of mammary stem/progenitor cells by PTEN/Akt/beta-catenin signaling. PLoS Biol..

[48] Hoey T., Yen W.C., Axelrod F., Basi J., Donigian L., Dylla S., Fitch-Bruhns M., Lazetic S., Park I.K., Sato A., Satyal S., Wang X., Clarke M.F., Lewicki J., Gurney A. (2009). DLL4 blockade inhibits tumor growth and reduces tumor-initiating cell frequency. Cell Stem Cell.

[49] Dierks C., Beigi R., Guo G.R., Zirlik K., Stegert M.R., Manley P., Trussell C., Schmitt-Graeff A., Landwerlin K., Veelken H., Warmuth M. (2008). Expansion of Bcr-Abl-positive leukemic stem cells is dependent on Hedgehog pathway activation. Cancer Cell.

[50] Zhao C., Chen A., Jamieson C.H., Fereshteh M., Abrahamsson A., Blum J., Kwon H.Y., Kim J., Chute J.P., Rizzieri D., Munchhof M., VanArsdale T., Beachy P.A., Reya T. (2009). Hedgehog signalling is essential for maintenance of cancer stem cells in myeloid leukaemia. Nature.

[51] Wicha M.S., Liu S., Dontu G. (2006). Cancer stem cells: An old idea -- a paradigm shift. Cancer Res..

[52] Liu S., Dontu G., Mantle I.D., Patel S., Ahn N.S., Jackson K.W., Suri P., Wicha M.S. (2006). Hedgehog signaling and Bmi-1 regulate self-renewal of normal and malignant human mammary stem cells. Cancer Res..

[53] Clement V., Sanchez P., de Tribolet N., Radovanovic I., Ruiz i Altaba A. (2007). HEDGEHOG-GLI1 signaling regulates human glioma growth, cancer stem cell self-renewal, and tumorigenicity. Curr. Biol..

[54] Varnat F., Duquet A., Malerba M., Zbinden M., Mas C., Gervaz P., Ruiz i Altaba A. (2009). Human colon cancer epithelial cells harbour active HEDGEHOG-GLI signalling that is essential for tumour growth, recurrence, metastasis and stem cell survival and expansion. EMBO Mol. Med..

[55] Dean M., Fojo T., Bates S. (2005). Tumour stem cells and drug resistance. Nat. Rev. Cancer.

[56] Bonnet D., Dick J.E. (1997). Human acute myeloid leukemia is organized as a hierarchy that originates from a primitive hematopoietic cell. Nat. Med..

[57] Lapidot T., Sirard C., Vormoor J., Murdoch B., Hoang T., Caceres-Cortes J., Minden M., Paterson B., Caligiuri M.A., Dick J.E. (1994). A cell initiating human acute myeloid leukaemia after transplantation into SCID mice. Nature.

[58] Al-Hajj M., Wicha M.S., Benito-Hernandez A., Morrison S.J., Clarke M.F. (2003). Prospective identification of tumorigenic breast cancer cells. Proc. Natl. Acad. Sci. USA.

[59] Singh S.K., Hawkins C., Clarke I.D., Squire J.A., Bayani J., Hide T., Henkelman R.M., Cusimano M.D., Dirks P.B. (2004). Identification of human brain tumour initiating cells. Nature.

[60] Ricci-Vitiani L., Lombardi D.G., Pilozzi E., Biffoni M., Todaro M., Peschle C., De Maria R. (2007). Identification and expansion of human colon-cancer-initiating cells. Nature.

[61] O'Brien C.A., Pollett A., Gallinger S., Dick J.E. (2007). A human colon cancer cell capable of initiating tumour growth in immunodeficient mice. Nature.

[62] Dalerba P., Dylla S.J., Park I.K., Liu R., Wang X., Cho R.W., Hoey T., Gurney A., Huang E.H., Simeone D.M., Shelton A.A., Parmiani G., Castelli C., Clarke M.F. (2007). Phenotypic characterization of human colorectal cancer stem cells. Proc. Natl. Acad. Sci. USA.

[63] Prince M.E., Sivanandan R., Kaczorowski A., Wolf G.T., Kaplan M.J., Dalerba P., Weissman I.L., Clarke M.F., Ailles L.E. (2007). Identification of a subpopulation of cells with cancer stem cell properties in head and neck squamous cell carcinoma. Proc. Natl. Acad. Sci. USA.

[64] Li C., Heidt D.G., Dalerba P., Burant C.F., Zhang L., Adsay V., Wicha M., Clarke M.F., Simeone D.M. (2007). Identification of pancreatic cancer stem cells. Cancer Res..

[65] Hermann P.C., Huber S.L., Herrler T., Aicher A., Ellwart J.W., Guba M., Bruns C.J., Heeschen C. (2007). Distinct populations of cancer stem cells determine tumor growth and metastatic activity in human pancreatic cancer. Cell Stem Cell.

[66] Schatton T., Murphy G.F., Frank N.Y., Yamaura K., Waaga-Gasser A.M., Gasser M., Zhan Q., Jordan S., Duncan L.M., Weishaupt C., Fuhlbrigge R.C., Kupper T.S., Sayegh M.H., Frank M.H. (2008). Identification of cells initiating human melanomas. Nature.

[67] Wu C., Wei Q., Utomo V., Nadesan P., Whetstone H., Kandel R., Wunder J.S., Alman B.A. (2007). Side population cells isolated from mesenchymal neoplasms have tumor initiating potential. Cancer Res..

[68] Yang Z.F., Ho D.W., Ng M.N., Lau C.K., Yu W.C., Ngai P., Chu P.W., Lam C.T., Poon R.T., Fan S.T. (2008). Significance of CD90+ cancer stem cells in human liver cancer. Cancer Cell.

[69] Eramo A., Lotti F., Sette G., Pilozzi E., Biffoni M., Di Virgilio A., Conticello C., Ruco L., Peschle C., De Maria R. (2008). Identification and expansion of the tumorigenic lung cancer stem cell population. Cell Death Differ..

[70] Collins A.T., Berry P.A., Hyde C., Stower M.J., Maitland N.J. (2005). Prospective identification of tumorigenic prostate cancer stem cells. Cancer Res..

[71] Curley M.D., Therrien V.A., Cummings C.L., Sergent P.A., Koulouris C.R., Friel A.M., Roberts D.J., Seiden M.V., Scadden D.T., Rueda B.R., Foster R. (2009). CD133 expression defines a tumor initiating cell population in primary human ovarian cancer. Stem Cells.

[72] Quintana E., Shackleton M., Foster H.R., Fullen D.R., Sabel M.S., Johnson T.M., Morrison S.J. (2010). Phenotypic heterogeneity among tumorigenic melanoma cells from patients that is reversible and not hierarchically organized. Cancer Cell.

[73] Kern S.E., Shibata D. (2007). The fuzzy math of solid tumor stem cells: A perspective. Cancer Res..

[74] Nakamura M., Okano H., Blendy J.A., Montell C. (1994). Musashi, a neural RNA-binding protein required for Drosophila adult external sensory organ development. Neuron.

[75] Okabe M., Sawamoto K., Imai T., Sakakibara S., Yoshikawa S., Okano H. (1997). Intrinsic and extrinsic determinants regulating cell fate decision in developing nervous system. Dev. Neurosci..

[76] Fujimoto K., Beauchamp R.D., Whitehead R.H. (2002). Identification and isolation of candidate human colonic clonogenic cells based on cell surface integrin expression. Gastroenterology.

[77] Batlle E., Henderson J.T., Beghtel H., van den Born M.M., Sancho E., Huls G., Meeldijk J., Robertson J., van de Wetering M., Pawson T., Clevers H. (2002). Beta-catenin and TCF mediate cell positioning in the intestinal epithelium by controlling the expression of EphB/ephrinB. Cell.

[78] van de Wetering M., Sancho E., Verweij C., de Lau W., Oving I., Hurlstone A., van der Horn K., Batlle E., Coudreuse D., Haramis A.P., Tjon-Pon-Fong M., Moerer P., van den Born M., Soete G., Pals S., Eilers M., Medema R., Clevers H. (2002). The beta-catenin/TCF-4 complex imposes a crypt progenitor phenotype on colorectal cancer cells. Cell.

[79] Sangiorgi E., Capecchi M.R. (2008). Bmi1 is expressed *in vivo* in intestinal stem cells. Nat. Genet..

[80] Du J., Li Y., Li J., Zheng J. (2010). Polycomb group protein Bmi1 expression in colon cancers predicts the survival. Med. Oncol..

[81] Sato T., Vries R.G., Snippert H.J., van de Wetering M., Barker N., Stange D.E., van Es J.H., Abo A., Kujala P., Peters P.J., Clevers H. (2009). Single Lgr5 stem cells build crypt-villus structures *in vitro* without a mesenchymal niche. Nature.

[82] Uchida H., Yamazaki K., Fukuma M., Yamada T., Hayashida T., Hasegawa H., Kitajima M., Kitagawa Y., Sakamoto M. (2010). Overexpression of leucine-rich repeat-containing G protein-coupled receptor 5 in colorectal cancer. Cancer Sci..

[83] Kemper K., Sprick M.R., de Bree M., Scopelliti A., Vermeulen L., Hoek M., Zeilstra J., Pals S.T., Mehmet H., Stassi G., Medema J.P. (2010). The AC133 epitope, but not the CD133 protein, is lost upon cancer stem cell differentiation. Cancer Res..

[84] Yin S., Li J., Hu C., Chen X., Yao M., Yan M., Jiang G., Ge C., Xie H., Wan D., Yang S., Zheng S., Gu J. (2007). CD133 positive hepatocellular carcinoma cells possess high capacity for tumorigenicity. Int. J. Cancer.

[85] Todaro M., Alea M.P., Di Stefano A.B., Cammareri P., Vermeulen L., Iovino F., Tripodo C., Russo A., Gulotta G., Medema J.P., Stassi G. (2007). Colon cancer stem cells dictate tumor growth and resist cell death by production of interleukin-4. Cell Stem Cell.

[86] Cammareri P., Lombardo Y., Francipane M.G., Bonventre S., Todaro M., Stassi G. (2008). Isolation and culture of colon cancer stem cells. Methods Cell Biol..

[87] Horst D., Kriegl L., Engel J., Kirchner T., Jung A. (2008). CD133 expression is an independent prognostic marker for low survival in colorectal cancer. Br. J. Cancer.

[88] Horst D., Kriegl L., Engel J., Jung A., Kirchner T. (2009). CD133 and nuclear beta-catenin: The marker combination to detect high risk cases of low stage colorectal cancer. Eur. J. Cancer.

[89] Horst D., Kriegl L., Engel J., Kirchner T., Jung A. (2009). Prognostic significance of the cancer stem cell markers CD133, CD44, and CD166 in colorectal cancer. Cancer Invest..

[90] Du L., Wang H., He L., Zhang J., Ni B., Wang X., Jin H., Cahuzac N., Mehrpour M., Lu Y., Chen Q. (2008). CD44 is of functional importance for colorectal cancer stem cells. Clin. Cancer Res..

[91] Huang E.H., Hynes M.J., Zhang T., Ginestier C., Dontu G., Appelman H., Fields J.Z., Wicha M.S., Boman B.M. (2009). Aldehyde dehydrogenase 1 is a marker for normal and malignant human colonic stem cells (SC) and tracks SC overpopulation during colon tumorigenesis. Cancer Res..

[92] Van Cutsem E., Kohne C.H., Hitre E., Zaluski J., Chang Chien C.R., Makhson A., D'Haens G., Pinter T., Lim R., Bodoky G., Roh J.K., Folprecht G., Ruff P., Stroh C., Tejpar S., Schlichting M., Nippgen J., Rougier P. (2009). Cetuximab and chemotherapy as initial treatment for metastatic colorectal cancer. N. Engl. J. Med..

[93] Lenz H.J. (2008). First-line combination treatment of colorectal cancer with hepatic metastases: Choosing a targeted agent. Cancer Treat. Rev..

[94] Los M., Roodhart J.M., Voest E.E. (2007). Target practice: Lessons from phase III trials with bevacizumab and vatalanib in the treatment of advanced colorectal cancer. Oncologist.

[95] Takahashi Y., Ellis L.M., Mai M. (2003). The angiogenic switch of human colon cancer occurs simultaneous to initiation of invasion. Oncol. Rep..

[96] Sokol C.L., Barton G.M., Farr A.G., Medzhitov R. (2008). A mechanism for the initiation of allergen-induced T helper type 2 responses. Nat. Immunol..

[97] Francipane M.G., Alea M.P., Lombardo Y., Todaro M., Medema J.P., Stassi G. (2008). Crucial role of interleukin-4 in the survival of colon cancer stem cells. Cancer Res..

[98] Blattman J.N., Greenberg P.D. (2004). Cancer immunotherapy: A treatment for the masses. Science.

[99] Koch U., Krause M., Baumann M. (2010). Cancer stem cells at the crossroads of current cancer therapy failures--radiation oncology perspective. Semin. Cancer Biol..

[100] Baumann M., Krause M., Hill R. (2008). Exploring the role of cancer stem cells in radioresistance. Nat. Rev. Cancer.

[101] Galon J., Costes A., Sanchez-Cabo F., Kirilovsky A., Mlecnik B., Lagorce-Pages C., Tosolini M., Camus M., Berger A., Wind P., Zinzindohoue F., Bruneval P., Cugnenc P.H., Trajanoski Z., Fridman W.H., Pages F. (2006). Type, density, and location of immune cells within human colorectal tumors predict clinical outcome. Science.

[102] Mattarollo S.R., Kenna T., Nieda M., Nicol A.J. (2006). Chemotherapy pretreatment sensitizes solid tumor-derived cell lines to V alpha 24+ NKT cell-mediated cytotoxicity. Int. J. Cancer.

[103] Todaro M., D'Asaro M., Caccamo N., Iovino F., Francipane M.G., Meraviglia S., Orlando V., La Mendola C., Gulotta G., Salerno A., Dieli F., Stassi G. (2009). Efficient killing of human colon cancer stem cells by gammadelta T lymphocytes. J. Immunol..

[104] Hao J., Wu X., Xia S., Li Z., Wen T., Zhao N., Wu Z., Wang P., Zhao L., Yin Z. (2010). Current progress in gammadelta T-cell biology. Cell Mol. Immunol..

